# Factors Associated With Online Patient-Provider Communications Among Cancer Survivors in the United States During COVID-19: Cross-sectional Study

**DOI:** 10.2196/44339

**Published:** 2023-05-22

**Authors:** Jiyeong Kim, Eleni Linos, Debra A Fishman, Melanie S Dove, Jeffrey S Hoch, Theresa H Keegan

**Affiliations:** 1 Department of Public Health Sciences School of Medicine University of California, Davis Davis, CA United States; 2 Stanford Center for Digital Health Stanford Medicine Stanford, CA United States; 3 Program for Clinical Research & Technology, Department of Dermatology School of Medicine Stanford University Stanford, CA United States; 4 Health Management and Education UC Davis Health Cardiac Rehabilitation Davis, CA United States; 5 Division of Health Policy and Management Department of Public Health Sciences University of California, Davis Davis, CA United States; 6 Division of Health Policy and Management, Department of Public Health Sciences Center for Healthcare Policy and Research University of California, Davis Davis, CA United States; 7 Division of Hematology and Oncology UC Davis Comprehensive Cancer Center Sacramento, CA United States

**Keywords:** online patient-provider communication, cancer survivor, COVID-19, telehealth, eHealth activities, telemedicine, eHealth, e-health, patient provider, online communication, patient-physician, national survey, sociodemographic, oncology, cancer

## Abstract

**Background:**

Online patient-provider communication (OPPC) is crucial in enhancing access to health information, self-care, and related health outcomes among cancer survivors. The necessity of OPPC increased during SARS-CoV-2/COVID-19, yet investigations in vulnerable subgroups have been limited.

**Objective:**

This study aims to assess the prevalence of OPPC and sociodemographic and clinical characteristics associated with OPPC among cancer survivors and adults without a history of cancer during COVID-19 versus pre–COVID-19.

**Methods:**

Nationally representative cross-sectional survey data (Health Information National Trends Survey 5, 2017-2020) were used among cancer survivors (N=1900) and adults without a history of cancer (N=13,292). COVID-19 data included data from February to June 2020. We calculated the prevalence of 3 types of OPPC, defined as using the email/internet, tablet/smartphone, or electronic health record (EHR) for patient-provider communication, in the past 12 months. To investigate the associations of sociodemographic and clinical factors with OPPC, multivariable-adjusted weighted logistic regression was performed to obtain odds ratios (ORs) and 95% CIs.

**Results:**

The average prevalence of OPPC increased from pre-COVID to COVID among cancer survivors (39.7% vs 49.7%, email/internet; 32.2% vs 37.9%, tablet/smartphone; 19.0% vs 30.0%, EHR). Cancer survivors (OR 1.32, 95% CI 1.06-1.63) were slightly more likely to use email/internet communications than adults without a history of cancer prior to COVID-19. Among cancer survivors, the email/internet (OR 1.61, 95% CI 1.08-2.40) and EHRs (OR 1.92, 95% CI 1.22-3.02) were more likely to be used during COVID-19 than pre–COVID-19. During COVID-19, subgroups of cancer survivors, including Hispanics (OR 0.26, 95% CI 0.09-0.71 vs non-Hispanic Whites) or those with the lowest income (US $50,000-<US $75,000: OR 6.14, 95% CI 1.99-18.92; ≥US $75,000: OR 0.42, 95% CI 1.56-11.28 vs <US $20,000), with no usual source of care (OR 6.17, 95% CI 2.12-17.99), or reporting depression (OR 0.33, 95% CI 0.14-0.78) were less likely to use email/internet, and those who were the oldest (age 35-49 years: OR 9.33, 95% CI 2.18-40.01; age 50-64 years: OR 3.58, 95% CI 1.20-10.70; age 65-74 years: OR 3.09, 95% CI 1.09-8.76 vs age≥75 years), were unmarried (OR 2.26, 95% CI 1.06-4.86), or had public/no health insurance (Medicare, Medicaid, or other: ORs 0.19-0.21 vs private) were less likely to use a tablet/smartphone to communicate with providers. Cancer survivors with a usual source of care (OR 6.23, 95% CI 1.66-23.39) or health care office visits in a year (ORs 7.55-8.25) were significantly more likely to use EHRs to communicate. Although it was not observed in cancer survivors, a lower education level was associated with lower OPPC among adults without a history of cancer during COVID-19.

**Conclusions:**

Our findings identified vulnerable subgroups of cancer survivors who were left behind in OPPC, which is increasingly becoming part of health care. These vulnerable subgroups of cancer survivors with lower OPPC should be helped through multidimensional interventions to prevent further inequities.

## Introduction

Online patient-provider communication (OPPC) refers to using online tools, including email/internet, tablets/smartphones, and mobile apps, for patient-provider communication [1]. Patient-provider communication is an essential element of cancer care and is associated with improved disease management, treatment adherence and quality, better health outcomes (eg, reduced mortality and mental distress), and superior health-related quality of life among cancer survivors [2-6]. Optimal OPPC has been found to have comparable benefits to face-to-face patient-provider communications among cancer survivors [7]. In addition, further benefits of OPPC among cancer survivors include increased access to health information, enhanced self-care ability, and an increased chance to be involved in health-related decision-making [8-10].

During the SARS-CoV-2/COVID-19 pandemic, the prevalence of poor mental health increased among cancer survivors [11-14]. Cancer survivors may have experienced a higher level of stress, fear, and psychological distress (eg, nervousness, worrying) due to delayed cancer care, fear of COVID-19 infection and poor health outcomes, or worry for cancer progression during COVID-19 than those without cancer [11,15-17]. Their unique situations would have required timely care and active communications with health providers to address health concerns and discuss care plans. Online-based health care became widely available in various health sectors during the early pandemic when in-person clinic visits were extremely limited owing to the pandemic [18-26]. Moreover, online-based care and communications will likely remain postpandemic for those who have medical conditions, because it became a major part of health care during the pandemic [27].

However, we do not know much about the adoption of online-based communications among cancer survivors during the early COVID-19 pandemic, although internet or digital device use behaviors in general US populations were assessed [28]. Given that OPPC use could also be a proxy of online-based care (eg, telehealth), which is only starting to be reported in some populations (eg, Medicare beneficiaries) [29,30], it is important to investigate subgroups who had low OPPC practice.

Previously, few studies have identified subgroups of cancer survivors who were vulnerable to OPPC before COVID-19 [7,31,32] and none, to the best of our knowledge, during the pandemic.

Before the COVID-19 pandemic, the adoption of and access to technology-based communication with providers was found to differ by some socioeconomic characteristics among cancer survivors. In a study by Jiang et al [7] using the national survey data (Health Information National Trends Survey [HINTS] 2008-2017), income, education, age, and health status were associated with OPPC via email, mobile platforms, and electronic health records (EHRs) among cancer survivors, yet the associations were inconsistent by year [7]. Two other studies, using HINTS (2003-2008 [31] and 2003-2018 [32]), found that young, highly educated, and metropolitan cancer survivors were more likely to email health care professionals. However, knowledge gaps still exist in OPPC practice among cancer survivors during COVID-19 compared to pre–COVID-19. Moreover, no studies have compared OPPC use in cancer survivors to the general population in prevalence and associations. Therefore, this study aimed to evaluate whether OPPC was higher among cancer survivors during COVID-19 than pre–COVID-19 and identify subgroups of cancer survivors with lower adoption of OPPC compared to those without a history of cancer during COVID-19.

## Methods

### Data Source

This study used nationally representative survey data from HINTS [33]. HINTS contains publicly available, self-administered, cross-sectional data collected by the National Cancer Institute (NCI). HINTS 5 Cycles 1-4 data from 2017 to 2020 were used for this study. HINTS 5 Cycles 1, 2, and 4 are single-mode mailed surveys that used a 2-stage sampling design, while HINTS 5 Cycle 3 is a double-mode design with a pilot push-to web survey in addition to the mailed survey. Remediated HINTS 5 Cycle 3 data were released in March 2021, and this study used the updated data. The survey questionnaires were distributed to noninstitutionalized civilians aged 18 years and older in the United States. HINTS 5 applied 2 stratified geographic addresses with areas of a high concentration of minority populations or a low concentration of minority populations, except for HINTS 5 Cycle 1. Cycle 1 used 3 stratified geographic addresses, adding the counties of Central Appalachia. The study followed Strengthening the Reporting of Observational Studies in Epidemiology (STROBE) guidelines [34]. The total number of survey respondents in HINTS 5 Cycles 1-4 was 16,092, and the 4-year average response rate was approximately 33.0% (n=3285, 32.4%, in Cycle 1; n=3504, 32.4%, in Cycle 2; n=5438, 30.3%, in Cycle 3; n=3865, 36.7%, in Cycle 4) [35]. Because we needed to combine the data from 4 survey cycles, we evaluated differences in variables across the cycles and the survey mode (mailed, push-to-web with paper return, push-to-web with web return) prior to merging the data. Because no critical discrepancies were identified in the variables of our interest by cycle, we merged the data from the 4 cycles, following the recommended analytic process provided by HINTS. We obtained 200 replicate weights, which were used to calculate SEs. Full sampling weights were applied for the sample to be nationally representative. The full sampling weight is intended to account for household-level base weight, nonresponse, person-level initial weight, and other biases [36]. Among the total respondents, excluding those who missed questions on a history of cancer (n=221, 1.4%), those who reported that they had ever been diagnosed with cancer were considered as cancer survivors after further excluding those with nonmelanoma skin cancer (N=1900) and the remaining (N=13,292) were considered as adults without a history of cancer.

### Outcomes

OPPC was measured using 3 types of communication behaviors, including the email/internet, tablet/smartphone, and EHR, as described previously [7]. Although the 3 types of OPPC might not be mutually exclusive, we used the following questions to measure different types and levels of participants’ behaviors in technology-based patient-provider communications: (1) “In the past 12 months, have you used email or the internet to communicate with a doctor or doctor's office?,” which required a basic level of technology literacy (email) and a technology-enabling environment (internet connection); (2) “Has your tablet or smartphone helped you in discussions with your health care provider?,” which demanded an advanced level of technology literacy (eg, live chatting, video visits) and digital device ownership (tablet, smartphone); and (3) “In the past 12 months, have you used your online medical record to securely message health care providers and staff?,” which additionally required some degree of engagement with the health care system. The responses were either yes or no, and those who answered yes were considered as practicing OPPC. The tablet/smartphone and EHR questions were only asked to those who owned tablet computers/smartphones or had used EHRs at least once in the past 12 months. In this study, those who did not have a tablet/smartphone or did not use EHRs once in the past 12 months were included in the no-OPPC groups using a tablet/smartphone or EHRs, respectively.

### Covariates

#### Sociodemographic Characteristics

We used the social determinants of the health conceptual framework from Healthy People 2030 [37] to choose sociodemographic factors as independent variables in this study: age (18-34, 35-49, 50-64, 65-74, ≥75 years), birth gender (male, female), race/ethnicity (non-Hispanic White, non-Hispanic Black/African American, Hispanic, non-Hispanic Asian, other), household income (<US $20,000, US $20,000-<US $35,000, US $35,000-<US $50,000, US $50,000-<US $75,000, ≥US $75,000), educational attainment (less than high school, high school graduate, some college, college graduate or more), marital status (married or living with a romantic partner as married vs not married, including divorced, widowed, separated, single/never been married), employment status (employed vs unemployed, including homemaker, student, retired, disabled), health insurance type (insured by employment, private insurance, Medicaid, Medicare, Tricare, Veterans Affairs [VA], Indian Health Services [IHS]), a usual source of care (yes, no), number of health care office visits (0, 1-4, 5-9), and rurality of residence (metropolitan, micropolitan, small town, rural). HINTS used the Urban-Rural Commuting Area (RUCA), which categorizes census tracts based on population density, urbanization, and commuting patterns developed by the United States Department of Agriculture to determine the rurality of residence of the respondents [38].

#### Clinical Characteristics

Clinical characteristics included general health status (excellent/very good/good, fair/poor), chronic medical conditions (diabetes, high blood pressure, heart disease, lung disease, depression), time since cancer diagnosis (<1 year, 2-5 years, 6-10 years, ≥11 years), psychological distress (little interest, hopelessness, nervousness, worrying), and cancer type the respondents were diagnosed with (breast, cervical, prostate, colon, lung, melanoma, bladder, bone, endometrial, head and neck, leukemia/blood, liver, lymphoma [Hodgkin and non-Hodgkin], oral, ovarian, pancreatic, pharyngeal, rectal, renal, stomach, multiple cancers). We recoded unknown and less prevalent cancer types, including bladder, bone, endometrial, head and neck, leukemia/blood, liver, lymphoma, oral, ovarian, pancreatic, pharyngeal, rectal, renal, and stomach cancer, as “other.”

### Statistical Analysis

We conducted survey-weighted descriptive analyses to demonstrate the sociodemographic and clinical characteristics of cancer survivors with frequency (n) and weighted percentage (%) during the COVID-19 (HINTS 5 Cycle 4, 2020) and pre–COVID-19 (HINTS 5 Cycles 1-3, 2017-2019) periods. Of note, the Cycle 4 questionnaires were collected from February to June 2020. Survey-weighted descriptive analyses were also performed to report the prevalence of 3 OPPC outcomes by sociodemographic and clinical factors among cancer survivors pre–COVID-19 and during COVID-19. We used multivariable-adjusted weighted logistic regression to obtain odds ratios (ORs) and associated 95% CIs to examine the associations of sociodemographic factors and clinical predictors with each OPPC outcome. The psychological distress measurements were converted to depression (little interest and hopelessness) or anxiety (nervousness and worrying) symptoms using the Patient Health Questionnaire-2 (PHQ-2) or General Anxiety Disorder-2 (GAD-2) scales, respectively, following their clinical cutoff (score≥3: symptom presents) [39]. Cancer survivors and adults without a history of cancer were analyzed in a model to compare the association of being a cancer survivor on each OPPC outcome after controlling for age, race/ethnicity, education, income, marital status, health insurance type, having a usual source of care, number of office visits, general health condition, chronic health condition (depression), and mental health (depression or anxiety symptoms). Because being a cancer survivor was associated with OPPC outcomes (email/internet use to communicate with providers, *P=*.035), we stratified cancer survivors and adults without a history of cancer to investigate the associations with sociodemographic and clinical factors. We developed 6 multivariable-adjusted weighted logistic regression models for 3 OPPC outcomes during COVID-19 and pre–COVID-19 among cancer survivors. Separately, 6 models were developed for adults without a history of cancer (Multimedia Appendix 1). Sociodemographic and clinical variables were included in a final model only if they were significantly associated with the outcome in univariable analyses (*P*<.05) or if they were considered a confounder for another covariate (eg, when the covariate effect estimate changed by more than 10%). Employment status was not reported in HINTS 5 Cycle 3, so it was not included in the models due to a huge portion of data unavailability (35.0%). For other covariates, the range of missingness varied from 0% to 13.3%, yet it was mostly less than 4.5%. To account for these missing data, which were considered suitable to impute, we applied a hot deck imputation method, which HINTS used to account for the nonresponse [36]. Adjustments for multiple testing were not performed, because this study was not confirmatory by design and we intended to avoid the potential risk of increasing type II errors [40,41]. Statistical significance was determined at *P*<.05 using SAS 9.4 (SAS Studio).

### Ethical Considerations

This study used the publicly available national survey data (HINTS). The study was a secondary analysis of survey data; human subjects were not involved, and identifiable information was not included. Given that the data were deidentified, the study was deemed exempt from review by the Institutional Review Board of the University of California, Davis.

## Results

### Description of Cancer Survivors

Of 1900 cancer survivors, 1444 (76.0%) were surveyed pre–COVID-19 (2017-2019) and 456 (24.0%) were surveyed during the COVID-19 pandemic (2020). There were no significant differences between the characteristics of the cancer survivors during the pre–COVID-19 and COVID-19 periods (Tables 1 and 2). Nearly half (n=289, 48.0%) were aged 65 years or older, 59.0% (n=272) were female, 79.0% (n=329) were non-Hispanic White, 63.0% (n=313) had some college education or more, 63.0% (n=228) were married, 62.0% (n=338) had public/government-aided health insurance, 84.0% (n=392) had a usual source of care, and 91.0% (n=420) had health care office visits at least once in a year. Clinically, 73.0% (n=322) reported that their general health status was good, while 56.0% (n=283) reported high blood pressure, 28.0% (n=149) had diabetes, 24.0% (n=111) had depression, and 12.0% (n=62) and 13.0% (n=60) reported that they had depressive and anxiety symptoms in the past 2 weeks, respectively. Nearly half of the cancer survivors (n=211, 46.0%) were 11 years or more from cancer diagnosis (Tables 1 and 2).

**Table 1:** Sociodemographic characteristics of cancer survivors (N=1900) pre–COVID-19 (2017-2019; HINTS^a^ 5 Cycles 1-3) and during COVID-19 (2020; HINTS 5 Cycle 4).

Characteristics			Pre–COVID-19^b^ (n=1444)^c^			During COVID-19^b^ (n=456)^c^	
		Frequency, n (%)		SE for weighted percentage	Frequency, n (%)		SE for weighted percentage
**Age (years)**							
	18-34	22 (5.7)		2.0	9 (2.3)		0.8
	35-49	99 (11.8)		1.6	31 (17.5)		3.7
	50-64	412 (31.8)		1.9	127 (32.8)		4.0
	65-74	477 (25.5)		1.6	155 (25.5)		2.6
	≥75	434 (25.2)		1.6	134 (22.0)		2.3
**Gender**							
	Female	875 (59.5)		2.0	272 (58.8)		4.0
	Male	569 (40.5)		2.0	184 (41.2)		4.0
**Race/ethnicity**							
	Non-Hispanic White	1057 (73.8)		2.0	329 (79.3)		2.6
	Non-Hispanic Black/African American	179 (11.0)		1.7	53 (8.3)		1.5
	Hispanic	120 (10.2)		1.5	53 (9.0)		2.2
	Non-Hispanic Asian	33 (2.0)		0.5	10 (1.5)		0.6
	Other	55 (3.1)		0.7	11 (1.8)		1.0
**Education**							
	Less than high school	88 (7.5)		1.6	39 (7.0)		1.7
	High school	315 (26.9)		2.0	104 (30.0)		3.1
	Some college	481 (40.1)		2.0	137 (39.9)		3.3
	College graduate or more	560 (25.6)		1.5	176 (23.1)		2.9
**Household income (US $)**							
	<20,000	284 (16.9)		1.7	100 (21.9)		3.0
	20,000-<35,000	242 (15.9)		1.4	73 (12.5)		2.2
	35,000-<50,000	194 (14.9)		2.3	72 (16.3)		2.6
	50,000-<75,000	285 (19.4)		1.7	78 (19.0)		3.1
	≥75,000	439 (32.8)		2.0	133 (30.2)		2.9
**Employment^d^**							
	Employed	228 (36.2)		2.5	126 (34.8)		3.7
	Unemployed	535 (63.8)		2.5	328 (65.2)		3.7
**Marital status**							
	Married	729 (59.6)		2.1	228 (63.3)		3.4
	Not married	715 (40.4)		2.1	228 (36.7)		3.4
**Rurality**							
	Metropolitan	1221 (83.6)		1.5	386 (78.7)		2.6
	Micropolitan	127 (9.9)		1.2	33 (10.9)		2.6
	Small town	56 (3.1)		0.6	18 (5.6)		2.1
	Rural	40 (3.4)		0.7	19 (4.7)		1.5
**Health insurance type**							
	Employment/private	359 (31.6)		2.1	118 (37.9)		3.7
	Medicare	570 (31.9)		1.7	179 (32.4)		2.9
	Medicaid	174 (16.6)		2.2	70 (16.7)		2.5
	Tricare, VA^e^, IHS^f^	173 (9.9)		1.2	40 (4.9)		1.1
	Other	168 (10.1)		1.0	49 (8.0)		2.1
**Usual source of care**							
	Yes	1205 (82.9)		1.4	392 (83.7)		3.2
	No	239 (17.1)		1.4	64 (16.3)		3.2
**Number of office visits in a year**							
	0	86 (7.4)		1.3	36 (9.5)		2.7
	1-4	791 (56.9)		2.5	234 (50.6)		4.0
	5-9	567 (35.8)		2.2	186 (39.9)		3.7

^a^HINTS: Health Information National Trends Survey.

^b^Missingness of covariates: pre–COVID-19 (age 2.1 %, gender 1.0%, race/ethnicity 11.9%, education 1.5%, income 13.0%, marital status 1.7%, health insurance type 4.4%, usual source of care 1.8%, general health status 1.5%, diabetes 2.8%, high blood pressure 2.4%, heart disease 1.6%, lung disease 1.7%, depression 2.6%, time since diagnosis 4.8%, cancer type 1.9%) and during COVID-19 (age 1.3 %, gender 0.7%, race/ethnicity 12.5%, education 3.9%, income 11.0%, marital status 2.9%, health insurance type 3.7%, usual source of care 3.3%, general health status 0.7%, diabetes 1.8%, high blood pressure 1.3%, heart disease 1.5%, lung disease 1.8%, depression 1.3, time since diagnosis 4.4%, cancer type 3.5%).

^c^Covariates with any missing values were imputed in the table.

^d^Employment data were not reported in Cycle 3; n=681 (35.8%) unavailable.

^e^VA: Veterans Affairs.

^f^IHS: Indian Health Services.

**Table 2:** Clinical characteristics of cancer survivors (N=1900) pre–COVID-19 (2017-2019; HINTS^a^ 5 Cycles 1-3) and during COVID-19 (2020; HINTS 5 Cycle 4).

Characteristics			Pre–COVID-19^b^ (n=1444)^c^			During COVID-19^b^ (n=456)^c^	
		Frequency, n (%)		SE for weighted percentage	Frequency, n (%)		SE for weighted percentage
**General health status**							
	Excellent/good	1073 (72.6)		1.9	322 (73.1)		3.0
	Fair/poor	371 (27.4)		1.9	134 (26.9)		3.0
**Chronic medical condition** **(ever told)**							
	Diabetes	415 (24.9)		1.8	149 (27.7)		3.0
	High blood pressure	860 (54.5)		2.1	283 (55.5)		3.5
	Heart disease	248 (15.6)		1.5	66 (11.6)		1.9
	Lung disease	243 (16.1)		1.2	106 (20.2)		2.9
	Depression	332 (22.7)		1.7	111 (24.0)		2.8
**Mental health (past 2 weeks)**							
	Depression symptoms	203 (16.0)		1.9	62 (11.6)		2.0
	Anxiety symptoms	168 (12.6)		1.4	60 (13.4)		2.2
**Time since diagnosis (years)**							
	<1	177 (13.3)		1.5	67 (16.1)		3.1
	2-5	313 (21.5)		1.8	87 (18.2)		2.9
	6-10	268 (16.6)		1.4	91 (19.6)		2.4
	≥11	686 (48.7)		2.0	211 (46.0)		3.7
**Cancer type**							
	Breast	282 (17.0)		1.4	88 (19.2)		3.3
	Cervical	96 (8.9)		1.4	36 (9.4)		2.5
	Prostate	173 (8.6)		1.0	61 (8.8)		1.5
	Colon	80 (5.4)		0.9	26 (4.6)		0.9
	Lung	37 (2.8)		0.6	12 (1.3)		0.5
	Melanoma	85 (5.1)		0.9	33 (10.9)		2.6
	Multiple	348 (23.7)		1.6	90 (17.4)		2.4
	Other	343 (28.5)		2.4	110 (28.4)		3.5

^a^HINTS: Health Information National Trends Survey.

^b^Missingness of covariates: pre–COVID-19 (age 2.1 %, gender 1.0%, race/ethnicity 11.9%, education 1.5%, income 13.0%, marital status 1.7%, health insurance type 4.4%, usual source of care 1.8%, general health status 1.5%, diabetes 2.8%, high blood pressure 2.4%, heart disease 1.6%, lung disease 1.7%, depression 2.6%, time since diagnosis 4.8%, cancer type 1.9%) and during COVID-19 (age 1.3 %, gender 0.7%, race/ethnicity 12.5%, education 3.9%, income 11.0%, marital status 2.9%, health insurance type 3.7%, usual source of care 3.3%, general health status 0.7%, diabetes 1.8%, high blood pressure 1.3%, heart disease 1.5%, lung disease 1.8%, depression 1.3, time since diagnosis 4.4%, cancer type 3.5%).

^c^Covariates with any missing values were imputed in the table.

### Prevalence of OPPC Among Cancer Survivors Compared to Adults Without a History of Cancer

**Figure 1:**
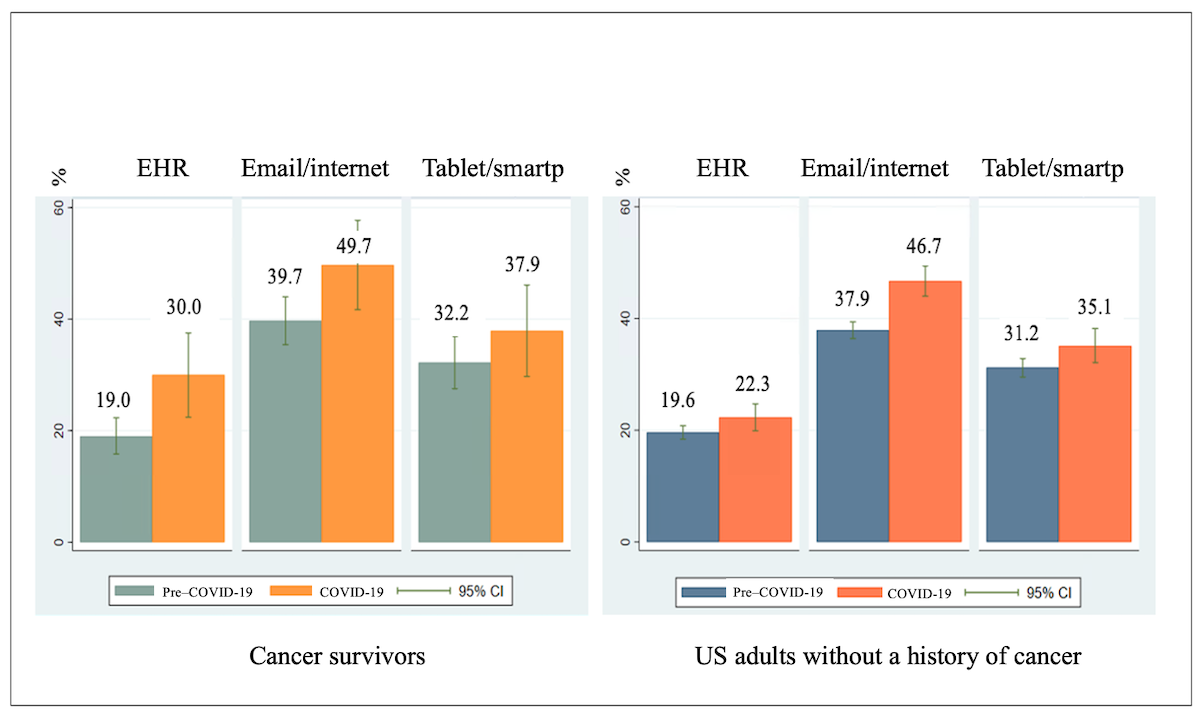
Prevalence of OPPC use. Pre–COVID-19 (2017-2019) and during COVID-19 (2020). The prevalence of OPPC use was presented as a weighted percentage. Cancer survivors (N=1900) and US adults without a history of cancer (N=13,292). EHR: electronic health record; OPPC: online patient-provider communication; smartp: smartphone.

The average prevalence of OPPC increased pre–COVID-19 to COVID-19 among cancer survivors: from 39.7% to 49.7% for email/internet use for communications with the provider/office, from 32.2% to 37.9% for tablet/smartphone use for discussions with providers, and from 19.0% to 30.0% for EHR use for messaging providers pre–COVID-19; see Figure 1. The average prevalence of OPPC among cancer survivors was similar to that among adults without a history of cancer pre–COVID-19 (approximate percentage, averaging out 3 OPPCs=29.0%) but was higher among cancer survivors during COVID-19. In multivariable models, cancer survivors were approximately 1.3 times as likely to use email/internet pre–COVID-19 than adults without a history of cancer (Table 3).

**Table 3:** Associations of a history of cancer with OPPC^a^ outcomes.

History of cancer	Pre–COVID-19^b^ (2017-2019; N=11,351), aOR^c,d^ (95% CI)			During COVID-19^b^ (2020), aOR^d^ (95% CI)		
	Email/internet (n=11,351)	Tablet/smartphone (n=10,759)	EHR^e^ (n=9751)	Email/internet (n=3568)	Tablet/smartphone (n=3554)	EHR (n=3541)
Yes	1.32 (1.06-1.63)^f^	1.21 (0.95-1.54)	0.98 (0.78-1.23)	1.28 (0.87-1.88)	1.20 (0.86-1.70)	1.39 (0.92-2.12)
No	Reference	Reference	Reference	Reference	Reference	Reference

^a^OPPC: online patient-provider communication.

^b^Total sample size: pre–COVID-19 (N=11,718) and during COVID-19 (N=3695).

^c^aOR: adjusted odds ratio.

^d^Adjusted by age, race/ethnicity, education, income, marital status, health insurance type, having a usual source of care, number of office visits, general health condition, chronic medical condition (depression), and mental health (depression or anxiety symptoms).

^e^EHR: electronic health record.

^f^*P*<.05.

### Prevalence of OPPC by Sociodemographic and Clinical Factors Pre–COVID-19 and During COVID-19

Tables 4 and 5 show the prevalence of OPPC by sociodemographic and clinical factors among cancer survivors before and during COVID-19. In general, cancer survivors who were younger than 65 years, were more educated (some college or more education), had a high income (US $50,000 or more), were married, were employed, were metropolitan residents, had private/employment-based insurance, had a usual source of care or health care office visits, had good general health status and chronic medical conditions (eg, depression), were recently diagnosed (<6 years) or diagnosed with breast cancer showed a high prevalence of OPPC than the average in both time periods. Although the prevalence of OPPC was similar between pre–COVID-19 and COVID-19 for most sociodemographic and clinical subgroups, there were some noticeable differences during COVID-19. Non-Hispanic White cancer survivors had higher-than- average prevalence in all 3 types of OPPC during COVID-19, while non-Hispanic Asians had higher OPPC before COVID-19.

**Table 4:** Prevalence of OPPC^a^ by sociodemographic factors among cancer survivors.

Characteristics			Pre–COVID-19 (2017-2019), weighted percentage (SE)							During COVID-19 (2020), weighted percentage (SE)				
		Email/internet		Tablet/smartphone		EHR^b^		Email/internet			Tablet/smartphone		EHR	
Average prevalence (%)			39.7 (2.2)		32.2 (2.4)		19.0 (1.6)		49.7 (4.0)			37.9 (4.1)		30.0 (3.8)
**Age (years)**														
	18-34	53.0 (21.8)^c^		56.8 (22.1)^c^		7.7 (5.2)		67.8 (23.9)^c^			40.6 (24.5)^c^		6.3 (7.0)	
	35-49	50.5 (6.4)^c^		36.4 (7.4)^c^		31.4 (6.1)^c^		58.8 (13.9)^c^			69.8 (13.2)^c^		21.5 (9.3)	
	50-64	46.4 (4.1)^c^		35.7 (3.9)^c^		19.9 (2.6)^c^		57.2 (7.1)^c^			50.0 (6.3)^c^		45.3 (7.5)^c^	
	65-74	39.1 (3.3)		35.0 (3.2)^c^		20.3 (2.8)^c^		40.8 (5.2)			23.7 (5.1)		25.0 (4.9)	
	≥75	23.2 (3.1)		15.6 (2.5)		12.9 (2.4)		39.0 (6.6)			9.9 (3.1)		21.0 (6.6)	
**Gender**														
	Female	37.1 (2.6)		29.7 (2.5)		19.5 (2.1)^c^		46.2 (6.0)			41.6 (5.5)^c^		27.2 (4.6)	
	Male	43.6 (4.0)^c^		35.8 (4.1)^c^		18.4 (2.8)		54.8 (4.5)^c^			33.0 (6.3)		34.0 (5.2)^c^	
**Race/ethnicity**														
	Non-Hispanic White	40.3 (2.2)^c^		29.0 (2.1)		20.6 (1.8)^c^		53.5 (4.4)^c^			38.3 (4.7)^c^		31.4 (4.3)^c^	
	Non-Hispanic Black/African American	44.5 (10.8)^c^		43.9 (11.9)^c^		10.7 (3.7)		30.6 (7.8)			27.8 (8.1)		26.6 (7.9)	
	Hispanic	28.7 (7.0)		36.8 (8.4)^c^		14.4 (6.7)		35.2 (11.8)			43.5 (10.8)^c^		24.4 (11.6)	
	Non-Hispanic Asian	50.2 (11.9)^c^		50.3 (12.3)^c^		36.5 (15.6)^c^		35.0 (18.3)			35.2 (18.7)		18.1 (14.2)	
	Other	38.8 (14.3)		43.7 (15.6)^c^		16.1 (7.8)		59.6 (30.0)^c^			47.8 (35.7)^c^		17.2 (13.9)	
**Education**														
	Less than high school	29.9 (18.5)		36.4 (20.0)^c^		3.8 (3.0)		30.2 (13.0)			11.2 (6.3)		20.9 (12.0)	
	High school	25.8 (3.7)		24.4 (4.0)		11.8 (2.7)		44.2 (7.9)			37.2 (8.5)		19.0 (6.6)	
	Some college	42.5 (3.7)^c^		33.8 (3.6)^c^		17.2 (2.8)		46.2 (6.6)			38.7 (6.6)^c^		28.8 (5.7)	
	College graduate or more	52.4 (3.1)^c^		36.2 (2.9)^c^		32.7 (3.2)^c^		69.8 (4.2)^c^			46.1 (7.0)^c^		50.0 (7.2)^c^	
**Household income (US $)**														
	<20,000	17.9 (3.2)		20.4 (4.2)		8.2 (2.1)		27.7 (6.7)			30.7 (8.8)		16.0 (5.5)	
	20,000-<35,000	29.4 (4.4)		26.3 (4.7)		15.2 (3.3)		30.2 (9.3)			21.5 (6.8)		15.7 (4.8)	
	35,000-<50,000	42.3 (7.2)^c^		35.4 (10.6)^c^		17.9 (5.0)		48.7 (8.9)			28.2 (6.6)		28.5 (7.7)	
	50,000-<75,000	44.6 (4.8)^c^		37.7 (4.7)^c^		20.8 (3.7)^c^		65.1 (8.8)^c^			45.9 (10.4)^c^		29.3 (7.9)	
	≥75,000	51.8 (3.5)^c^		35.8 (3.7)^c^		25.4 (3.0)^c^		63.5 (7.1)^c^			48.7 (7.4)^c^		47.3 (8.5)^c^	
**Marital status**														
	Married	44.1 (2.4)^c^		34.0 (2.7)^c^		20.9 (2.1)^c^		54.1 (5.2)^c^			44.8 (5.5)^c^		34.7 (5.5)^c^	
	Not married	33.3 (4.0)		29.5 (4.2)		16.2 (2.4)		42.0 (6.4)			24.8 (4.2)		21.6 (4.9)	
**Employment**														
	Employed	49.4 (4.8)^c^		33.8 (4.8)^c^		21.8 (4.0)^c^		65.6 (7.4)^c^			59.7 (6.8)^c^		39.3 (8.0)^c^	
	Unemployed	31.0 (3.3)		31.0 (3.7)		16.1 (2.7)		41.1 (4.1)			26.3 (4.6)		24.9 (2.9)	
**Rurality**														
	Metropolitan	42.5 (2.4)^c^		34.0 (2.6)^c^		19.7 (1.9)^c^		51.2 (4.5)^c^			38.6 (4.5)^c^		30.7 (4.0)^c^	
	Micropolitan	28.0 (5.2)		20.4 (5.5)		16.9 (4.8)		51.1 (14.7)			35.2 (17.3)		43.5 (16.4)	
	Small town	26.6 (7.2)		30.4 (8.7)		14.7 (6.8)		10.7 (10.9)			34.5 (33.4)		10.5 (11.8)	
	Rural	16.2 (7.8)		23.8 (9.7)		12.7 (7.0)		68.2 (18.0)^c^			36.9 (19.5)		8.5 (5.6)	
**Health insurance**														
	Employment/private	55.9 (4.2)^c^		36.8 (4.2)^c^		23.8 (3.1)^c^		64.9 (7.3)^c^			65.6 (6.0)^c^		43.6 (8.0)^c^	
	Medicare	34.2 (2.8)		27.0 (2.7)		20.5 (2.5)^c^		39.2 (5.3)			17.2 (3.7)		22.6 (4.2)	
	Medicaid	31.4 (8.9)		37.5 (9.5)^c^		13.4 (4.3)		40.8 (10.7)			26.0 (7.7)		17.0 (6.8)	
	Tricare, VA^d^, IHS^e^	29.0 (5.3)		30.3 (5.2)		13.4 (4.4)		60.1 (12.9)^c^			29.8 (9.3)		32.0 (11.0)^c^	
	Other	30.3 (4.5)		26.7 (5.0)		14.7 (3.9)		35.1 (7.8)			18.9 (4.9)		23.2 (6.1)	
**Usual source of care**														
	Yes	42.3 (2.5)^c^		34.8 (2.7)^c^		20.9 (2.0)^c^		53.6 (4.1)^c^			37.8 (4.7)		34.2 (4.0)^c^	
	No	27.5 (4.5)		20.0 (4.0)		8.9 (2.4)		26.6 (11.8)			38.6 (14.9)^c^		5.7 (2.9)	
**Number of office visits in a year**														
	0	20.6 (6.6)		10.7 (4.9)		7.9 (6.3)		43.9 (16.3)			30.3 (19.5)		3.9 (2.7)	
	1-4	39.8 (2.9)^c^		30.5 (3.3)		16.0 (2.0)		47.7 (5.2)			37.6 (6.1)		31.9 (4.7)^c^	
	5-9	43.6 (3.4)^c^		39.7 (3.7)^c^		25.7 (3.1)^c^		53.7 (6.7)^c^			39.9 (6.8)^c^		33.5 (5.6)^c^	

^a^OPPC: online patient-provider communication.

^b^EHR: electronic health record.

^c^Prevalence is higher than the average.

^d^VA: Veterans Affairs.

^e^IHS: Indian Health Services.

**Table 5:** Prevalence of OPPC^a^ by clinical factors among cancer survivors.

Characteristics			Pre–COVID-19 (2017-2019), weighted percentage (SE)				During COVID-19 (2020), weighted percentage (SE)		
		Email/internet		Tablet/smartphone	EHR^b^	Email/internet		Tablet/smartphone	EHR
**General health** **status**									
	Excellent/good	42.6 (2.5)^c^		30.9 (2.8)	19.5 (2.0)^c^	54.9 (4.5)^c^		43.5 (4.5)^c^	32.0 (4.4)^c^
	Fair/poor	31.9 (3.8)		35.9 (4.4)^c^	17.9 (3.0)	35.4 (6.4)		23.0 (5.4)	24.7 (5.9)
**Chronic condition (ever diagnosed)**									
	Diabetes	35.5 (4.0)		29.1 (4.1)	18.4 (3.3)	47.7 (7.3)		32.6 (6.9)	32.5 (7.7)^c^
	High blood pressure	37.2 (2.6)		30.7 (2.4)	19.5 (2.2)^c^	51.6 (4.4)^c^		33.2 (5.7)	33.1 (5.1)^c^
	Heart disease	36.7 (4.9)		33.2 (5.1)^c^	20.1 (4.3)^c^	37.5 (8.4)		27.6 (7.4)	23.1 (6.3)
	Lung disease	32.9 (4.6)		30.2 (4.9)	18.2 (4.0)	43.6 (6.2)		33.6 (7.6)	30.0 (5.6)
	Depression	44.4 (3.9)^c^		38.1 (4.6)^c^	23.9 (4.0)^c^	38.4 (7.3)		38.9 (7.5)^c^	26.3 (5.9)
**Mental health (past 2 weeks)**									
	Depression symptoms	41.2 (8.1)^c^		38.3 (8.9)^c^	15.7 (4.4)	40.1 (9.3)		27.3 (10.0)	21.1 (7.8)
	Anxiety symptoms	42.3 (5.8)^c^		36.0 (5.8)^c^	20.4 (5.1)^c^	46.0 (9.5)		36.0 (9.7)	28.4 (8.4)
**Time since diagnosis (years)**									
	<1	43.9 (6.2)^c^		36.4 (6.6)^c^	19.4 (4.9)^c^	63.8 (10.2)^c^		54.8 (12.1)^c^	32.7 (10.1)^c^
	2-5	49.5 (5.5)^c^		43.7 (6.2)^c^	25.0 (4.3)^c^	47.2 (8.8)		35.0 (8.6)	30.2 (8.2)^c^
	6-10	39.4 (4.2)		29.0 (3.9)	22.5 (4.1)^c^	38.9 (9.3)		32.4 (9.4)	19.0 (5.3)
	≥11	34.3 (3.1)		27.0 (2.7)	14.9 (2.1)	50.3 (5.7)^c^		35.3 (5.4)	33.2 (5.9)^c^
**Cancer type**									
	Breast	39.9 (4.0)^c^		36.8 (4.5)^c^	23.6 (3.9)^c^	55.8 (9.1)^c^		52.2 (9.1)^c^	32.3 (8.1)^c^
	Cervical	41.7 (7.9)^c^		31.1 (7.8)	22.9 (7.0)^c^	49.7 (16.4)		39.4 (15.1)^c^	27.2 (13.8)
	Prostate	34.1 (5.2)		29.3 (4.9)	12.6 (3.5)	48.4 (11.3)		18.9 (7.1)	35.2 (10.3)^c^
	Colon	42.2 (10.9)^c^		50.2 (11.2)^c^	10.6 (8.1)	45.0 (12.9)		26.8 (10.7)	24.6 (10.0)
	Lung	19.8 (8.5)		11.2 (6.8)	7.0 (3.6)	38.2 (22.2)		41.4 (21.5)^c^	7.6 (6.3)
	Melanoma	45.5 (8.9)^c^		20.8 (5.9)	23.6 (7.9)^c^	52.8 (14.1)^c^		40.0 (17.0)^c^	31.3 (14.0)^c^
	Multiple	43.0 (4.5)^c^		31.5 (3.7)	22.0 (3.5)^c^	49.3 (9.7)		20.8 (5.6)	29.6 (7.8)
	Other	38.6 (5.2)		32.4 (5.6)^c^	16.5 (2.8)	46.2 (8.2)		45.0 (9.3)^c^	29.3 (7.3)

^a^OPPC: online patient-provider communication.

^b^EHR: electronic health record.

^c^Prevalence is higher than the average.

### Sociodemographic and Clinical Factors Associated With OPPC Among Cancer Survivors Pre–COVID-19 vs COVID-19

Email/internet and EHR-based communications were 1.5-2 times as likely to be used during COVID-19 than pre–COVID-19 (email/internet: OR 1.61, 95% CI 1.08-2.40; EHR: OR 1.92, 95% CI 1.22-3.02).

Pre–COVID-19, younger age groups (18-74 years old) had nearly 2-9 times the odds of using the email/internet, tablet/smartphone, or EHR to communicate with providers compared to those 75 years or older (Tables 6-8). Cancer survivors with a higher annual income (US $20,000 or more) were 2-3.5 times as likely to communicate electronically with providers via the email/internet, tablet/smartphone, or EHR than those with less than US $20,000 of income. Those insured by private or employment-based plans had 2 times the odds of using email/internet for communications than those with public/government-supported insurance (Medicaid, Tricare/VA/IHS, other: ORs 0.41-0.49). Those who were recently diagnosed with cancer (2-5 years) were nearly 2 times as likely to use the email/internet, tablet/smartphone, or EHR for communications with providers/offices as those diagnosed more than 10 years ago (OR 2.02, 95% CI 1.23-3.33; OR 1.86, 95% CI 1.14-3.03; and OR 2.30, 95% CI 1.29-4.11, respectively). Those with a usual source of health care had 2.5 times (OR 2.55, 95% CI 1.21-5.38) the odds of using EHRs, and those who had health care office visits at least once had 4-6 times (ORs 4.46-5.91) the odds of using a tablet/smartphone to communicate with providers compared to those without a usual source of care or office visits. Breast cancer survivors were more likely to use a tablet/smartphone and EHRs than lung cancer survivors to communicate with providers.

During COVID-19, cancer survivors with a usual source of care had 6 times the odds of using email/internet (OR 6.17, 95% CI 2.12-17.99) or EHRs (OR 6.23, 95% CI 1.66-23.39) to communicate with providers/offices (Tables 6-8). Moreover, those who had health care office visits at least once in a year were 8 times as likely to use EHRs to send messages to the provider (1-4 times: OR 8.25, 95% CI 1.61-42.18; 5-9 times: OR 7.55, 95% CI 1.56-36.60) than those without any office visits. Hispanic cancer survivors (OR 0.26, 95% CI 0.09-0.71) were significantly less likely to use email/internet to communicate with providers/offices than their non-Hispanic White counterparts. Cancer survivors with more income (≥US $50,000 vs <US $20,000) had 4-6 times the odds of using email/internet for communications with providers/offices. Cancer survivors reporting a history of depression diagnosis were less likely to use email/internet to communicate with providers/offices (OR 0.33, 95% CI 0.14-0.78). The oldest individuals (≥75 years) were significantly less likely to use a tablet/smartphone to discuss with providers than their younger counterparts (35-74 years: ORs 3.09-9.33). Married cancer survivors were 2 times as likely to use a tablet/smartphone for communications (OR 2.26, 95% CI 1.06-4.86). Cancer survivors insured by Medicare (OR 0.21, 95% CI 0.08-0.54), Medicaid (OR 0.19, 95% CI 0.06-0.61), or other types of health plans (OR 0.20, 95% CI 0.07-0.58) were significantly less likely to discuss with providers via a tablet/smartphone than those with private or employment-based insurance.

**Table 6:** Associations of time period with OPPC^a^ among cancer survivors.

Time period	Email/internet, aOR^b,c^ (95% CI)	Tablet/smartphone, aOR^c^ (95% CI)	EHR^d^, aOR^c^ (95% CI)
During COVID-19 (2000)	1.61 (1.08-2.40)^e^	1.40 (0.90-2.20)	1.92 (1.22-3.02)^e^
Pre–COVID-19 (2017-2019)	Reference	Reference	Reference

^a^OPPC: online patient-provider communication.

^b^aOR: adjusted odds ratio.

^c^Adjusted for all the variables in the table.

^d^EHR: electronic health record.

^e^*P*<.05.

**Table 7:** Associations of sociodemographic factors with OPPC^a^ among cancer survivors pre–COVID-19 (2017-2019) and during COVID-19 (2020).

Factors			Pre–COVID-19 (N=1444), aOR^b,c^ (95% CI)				During COVID-19 (N=456), aOR^c^ (95% CI)		
		Email/internet (n=1411)		Tablet/smartphone (n=1307)	EHR^d^ (n=1229)	Email/internet (n=446)		Tablet/smartphone (n=441)	EHR (n=444)
**Age (years)**									
	18-34	7.43 (2.47-22.29)^e^		9.59 (3.03-30.35)^e^	0.87 (0.21-3.65)	5.38 (0.65-44.88)		1.04 (0.03-39.71)	0.40 (0.01-11.97)
	35-49	2.52 (1.18-5.39)^e^		2.85 (1.26-6.46)^e^	2.52 (1.03-6.19)^e^	3.53 (0.55-22.47)		9.33 (2.18-40.01)^e^	1.13 (0.18-7.14)
	50-64	2.30 (1.30-4.06)^e^		2.85 (1.62-5.01)^e^	1.47 (0.69-3.11)	1.74 (0.43-7.10)		3.58 (1.20-10.70)^e^	1.94 (0.38-9.82)
	65-74	2.16 (1.36-3.43)^e^		2.91 (1.81-4.66)^e^	1.53 (0.86-2.73)	1.25 (0.45-3.43)		3.09 (1.09-8.76)^e^	1.31 (0.42-4.13)
	≥75	Reference		Reference	Reference	Reference		Reference	Reference
**Race/ethnicity**									
	Non-Hispanic White	Reference		Reference	Reference	Reference		Reference	Reference
	Non-Hispanic Black/African American	1.37 (0.72-2.63)		1.87 (0.98-3.57)	0.58 (0.25-1.33)	0.64 (0.24-1.69)		1.16 (0.46-2.92)	1.04 (0.32-3.38)
	Hispanic	0.60 (0.29-1.27)		2.67 (0.49-2.79)	0.83 (0.28-2.43)	0.26 (0.09-0.71)^e^		1.14 (0.32-4.05)	0.47 (0.16-1.39)
	Non-Hispanic Asian	1.27 (0.51-3.13)		2.67 (0.93-7.64)	2.11 (0.66-6.70)	0.32 (0.07-1.40)		1.33 (0.17-10.78)	0.47 (0.07-3.31)
	Other	0.78 (0.34-1.82)		1.09 (0.38-3.11)	0.98 (0.30-3.23)	1.62 (0.30-8.89)		1.39 (0.17-11.41)	0.47 (0.08-2.62)
**Education**									
	Less than high school	Reference		Reference	Reference	Reference		Reference	Reference
	High school	0.99 (0.41-2.38)		0.65 (0.25-1.74)	2.37 (0.45-12.57)	0.67 (0.13-3.61)		2.54 (0.35-18.35)	0.48 (0.07-3.46)
	Some college	1.64 (0.71-3.78)		0.96 (0.41-2.24)	2.93 (0.59-14.65)	0.90 (0.18-4.60)		2.61 (0.33-20.58)	0.80 (0.12-5.37)
	College graduate or more	1.94 (0.78-4.81)		1.00 (0.41-2.47)	6.24 (1.22-32.05)^e^	1.75 (0.40-7.62)		2.88 (0.34-24.23)	1.76 (0.27-11.38)
**Household income (US $)**									
	<20,000	Reference		Reference	Reference	Reference		Reference	Reference
	20,000-<35,000	2.03 (1.00-4.11)^e^		2.41 (1.07-5.40)^e^	1.79 (0.76-4.23)	2.08 (0.61-7.07)		1.04 (0.31-3.55)	0.79 (0.21-2.91)
	35,000-<50,000	3.40 (1.70-6.82)^e^		2.88 (1.22-6.80)^e^	2.14 (0.94-4.91)	2.69 (0.77-9.38)		0.66 (0.20-2.16)	1.51 (0.38-6.03)
	50,000-<75,000	3.26 (1.69-6.29)^e^		3.22 (1.56-6.66)^e^	2.20 (1.06-4.56)^e^	6.14 (1.99-18.92)^e^		2.07 (0.34-3.33)	1.67 (0.53-5.23)
	≥75,000	3.55 (1.82-6.90)^e^		3.03 (1.46-6.28)^e^	2.36 (1.05-5.31)^e^	4.20 (1.56-11.28)^e^		0.99 (0.32-3.09)	1.59 (0.52-4.85)
**Marital status**									
	Married	1.10 (0.72-1.69)		1.20 (0.80-1.81)	0.83 (0.52-1.32)	0.88 (0.46-1.67)		2.26 (1.06-4.86)^e^	1.09 (0.54-2.20)
	Not married	Reference		Reference	Reference	Reference		Reference	Reference
**Health insurance type**									
	Employment/private	Reference		Reference	Reference	Reference		Reference	Reference
	Medicare	0.65 (0.38-1.10)		0.99 (0.54-1.83)	1.19 (0.58-2.43)	0.47 (0.16-1.35)		0.21 (0.08-0.54)^e^	0.41 (0.13-1.35)
	Medicaid	0.48 (0.25-0.91)^e^		1.01 (0.49-2.11)	0.88 (0.37-2.11)	0.83 (0.24-2.90)		0.19 (0.06-0.61)^e^	0.36 (0.11-1.21)
	Tricare, VA^f^, IHS^g^	0.41 (0.21-0.80)^e^		1.05 (0.53-2.09)	0.61 (0.26-1.44)	1.42 (0.39-5.26)		0.69 (0.21-2.29)	0.89 (0.21-3.78)
	Other	0.49 (0.27-0.89)^e^		0.88 (0.43-1.79)	0.71 (0.29-1.75)	0.34 (0.09-1.37)		0.20 (0.07-0.58)^e^	0.34 (0.010-1.21)
**Usual source of care**									
	Yes	1.58 (0.88-2.84)		1.58 (0.91-2.76)	2.55 (1.21-5.38)^e^	6.17 (2.12-17.99)^e^		0.98 (0.26-3.69)	6.23 (1.66-23.39)^e^
	No	Reference		Reference	Reference	Reference		Reference	Reference
**Number of office visits** **in a year**									
	0	Reference		Reference	Reference	Reference		Reference	Reference
	1-4	2.05 (0.73-5.77)		4.46 (1.49-13.37)^e^	1.98 (0.51-7.60)	0.83 (0.26-2.63)		2.15 (0.50-9.25)	8.25 (1.61-42.18)^e^
	5-9	2.55 (0.90-7.22)		5.91 (1.94-17.97)^e^	2.85 (0.67-12.02)	1.18 (0.35-3.97)		2.32 (0.52-10.34)	7.55 (1.56-36.60)^e^

^a^OPPC: online patient-provider communication.

^b^aOR: adjusted odds ratio.

^c^Adjusted for all the variables in the table.

^d^EHR: electronic health record.

^e^*P*<.05.

^f^VA: Veterans Affairs.

^g^IHS: Indian Health Services.

**Table 8:** Associations of clinical factors with OPPC^a^ among cancer survivors pre–COVID-19 (2017-2019) and during COVID-19 (2020).

Factors			Pre–COVID-19 (N=1444), aOR^b,c^ (95% CI)				During COVID-19 (N=456), aOR^c^ (95% CI)		
		Email/internet (n=1411)		Tablet/smartphone (n=1307)	EHR^d^ (n=1229)	Email/internet (n=446)		Tablet/smartphone (n=441)	EHR (n=444)
**General health status**									
	Excellent/good	1.36 (0.87-2.12)		0.79 (0.49-1.28)	0.81 (0.45-1.48)	1.52 (0.64-3.63)		1.94 (0.82-4.60)	0.84 (0.35-2.00)
	Fair/poor	Reference		Reference	Reference	Reference		Reference	Reference
**Chronic condition**									
	Depression	1.46 (0.93-2.29)		1.43 (0.88-2.32)	1.43 (0.80-2.57)	0.33 (0.14-0.78)^e^		1.59 (0.55-4.55)	0.73 (0.32-1.70)
	No depression	Reference		Reference	Reference	Reference		Reference	Reference
**Mental health (past 2 weeks)**									
	Depression symptoms	1.35 (0.69-2.66)		1.10 (0.56-2.17)	0.87 (0.39-1.92)	0.99 (0.24-4.10)		0.52 (0.14-2.00)	0.41 (0.07-2.29)
	No symptoms	Reference		Reference	Reference	Reference		Reference	Reference
	Anxiety symptoms	1.23 (0.61-2.48)		1.10 (0.54-2.23)	1.24 (0.53-2.88)	2.21 (0.51-9.61)		1.52 (0.29-7.93)	2.14 (0.53-8.62)
	No symptoms	Reference		Reference	Reference	Reference		Reference	Reference
**Time since diagnosis** **(years)**									
	<1	1.56 (0.88-2.77)		1.49 (0.81-2.74)	1.36 (0.65-2.84)	1.26 (0.47-3.40)		2.15 (0.69-6.69)	0.88 (0.24-3.15)
	2-5	2.02 (1.23-3.33)^e^		1.86 (1.14-3.03)^e^	2.30 (1.29-4.11)^e^	0.97 (0.40-2.39)		0.54 (0.18-1.63)	1.17 (0.50-2.70)
	6-10	1.21 (0.76-1.92)		0.99 (0.60-1.61)	1.83 (0.97-3.43)	0.47 (0.20-1.09)		0.59 (0.26-1.35)	0.42 (0.15-1.18)
	≥11	Reference		Reference	Reference	Reference		Reference	Reference
**Cancer type**									
	Breast	Reference		Reference	Reference	Reference		Reference	Reference
	Cervical	0.94 (0.40-2.21)		0.61 (0.26-1.43)	1.28 (0.51-3.22)	0.90 (0.26-3.10)		0.41 (0.13-1.30)	1.58 (0.31-8.22)
	Prostate	1.01 (0.51-1.97)		0.79 (0.41-1.53)	0.43 (0.17-1.09)	1.17 (0.38-3.57)		0.26 (0.09-0.77)^e^	1.65 (0.48-5.69)
	Colon	1.08 (0.45-2.57)		1.47 (0.60-3.59)	0.40 (0.10-1.66)	1.74 (0.42-7.21)		0.87 (0.22-3.45)	1.60 (0.36-7.01)
	Lung	0.41 (0.14-1.20)		0.14 (0.04-0.47)^e^	0.26 (0.08-0.86)^e^	1.68 (0.25-11.27)		3.21 (0.59-17.42)	0.26 (0.02-2.92)
	Melanoma	0.99 (0.39-2.49)		0.41 (0.17-1.00)	0.85 (0.31-2.33)	0.97 (0.24-3.92)		0.39 (0.08-1.98)	0.82 (0.18-3.71)
	Multiple	1.81 (0.97-3.36)		0.99 (0.56-1.78)	1.18 (0.63-2.22)	1.07 (0.32-3.65)		0.42 (0.14-1.28)	1.14 (0.31-4.20)
	Other	0.88 (0.48-1.59)		0.62 (0.35-1.07)	0.72 (0.38-1.36)	0.90 (0.32-2.53)		0.61 (0.24-1.58)	1.26 (0.38-4.18)

^a^OPPC: online patient-provider communication.

^b^aOR: adjusted odds ratio.

^c^Adjusted for all the variables in the table.

^d^EHR: electronic health record.

^e^*P*<.05.

### Cancer Survivors vs Adults Without a History of Cancer

Among cancer survivors (Tables 6-8) and adults without a history of cancer (Multimedia Appendix 1), those with a usual source of care were 2-6 times as likely to use OPPC than those without a source pre–COVID-19 and during COVID-19. Among those without a history of cancer in both time periods, those who were more educated were 2-6 times and those who reported depression were 1.5-2 times as likely to use OPPC (Multimedia Appendix 1). However, among cancer survivors, we did not observe associations with education and found that depression was inversely associated with OPPC.

## Discussion

### Principal Findings

Using nationally representative survey data in the United States from 2017 to 2020, we identified that having a usual source of care or health care office visits is strongly associated with 3 types of OPPC, and different sociodemographic and clinical characteristics were associated with OPPC among cancer survivors and adults without a history of cancer during the pre–COVID-19 and COVID-19 periods. Cancer survivors were more likely to use email/internet to communicate with providers than those without a history of cancer prior to the COVID-19 pandemic, yet no difference was found during the early pandemic. However, OPPC use was higher during COVID-19 than pre–COVID-19 among cancer survivors. During COVID-19, subgroups of cancer survivors were less likely to use OPPC, including the oldest cancer survivors (≥75 years), who were Hispanic, had the lowest income, were unmarried, had no usual source of care or no visits to health providers, had public/no health insurance, or reported having depression. However, a lower education level was associated with lower OPPC among adults without a history of cancer during COVID-19. Our findings identified vulnerable subgroups of cancer survivors who were left behind in OPPC, which is increasingly becoming part of health care [19-21,24].

During COVID-19, but not prior to the pandemic, cancer survivors who were not married or had Medicare, Medicaid, or other health plans, including no insurance, were significantly less likely to use a tablet/smartphone to communicate with providers. Our marital status findings are consistent with prior studies that have found that individuals living with a spouse or partner are more likely to perform healthy behaviors (eg, a higher success rate of quitting tobacco [42,43]). Differences by health insurance could be related to the surge in telehealth use among those with private/employment-based insurance when major insurance companies started reimbursement for telehealth services in early 2020 [44]. The Centers for Medicare and Medicaid Services (CMS) also expanded health care professionals’ role to provide telemedicine to increase telehealth access and its use, including telephone/audio-only or e-visits [45-47]. However, the CMS’s effort to create an enabling environment for telehealth use might not have been enough for cancer survivors with Medicare or Medicaid to increase their use of mobile devices (eg, tablets/smartphones) for communication with providers compared to those with private/employment-based insurance.

Although racial/ethnic differences were not observed among cancer survivors prior to COVID-19 in this study and previously [7,31,32], we observed that Hispanic cancer survivors were significantly less likely to have online communications with providers/offices via email/internet than their non-Hispanic White counterparts during COVID-19. Early in the pandemic, Hispanic populations had higher rates of COVID-19–related hospitalization, intensive care unit admission, or in-hospital death [48,49], which could have been related to a higher prevalence of chronic diseases [50] or having more unmet health care needs [51]. In our study, chronic disease prevalence was not significantly different between racial/ethnic groups, but we were unable to account for unmet health care needs, other than lacking a usual source of care, that could have resulted in less use of online tools to communicate with providers.

Before COVID-19, cancer survivors aged ≥75 years were least likely to practice OPPC via email, the internet, a tablet, or a smartphone. This was also observed among adults without a history of cancer in this study, which aligns with the previous literature [28]. Prior studies suggest that adults aged 65 years and older had less interest in exchanging medical information online with providers [52], less frequently used social media for health communication [53], and less frequently used the internet to search for health information [54] compared to younger generations. This could be potentially due to lower eHealth literacy or higher computer stress among the oldest (≥70 years) compared with younger individuals [55-57]. Older individuals have poorer COVID-19 outcomes [58] and a higher level of fear of COVID-19 [59]; hence, their demands for OPPC might have been high to avoid possible exposure during our study period, yet the barriers noted before could have limited their use of OPPC. In addition, low income was significantly associated with lower OPPC among cancer survivors before COVID-19, consistent with low income being strongly associated with low health technology use in the general population [52,55]. Specifically, low-income older adults designated a lack of financial resources as a barrier to technology access and ownership [60]. However, these strong associations with low income in OPPC were less evident among cancer survivors during COVID-19, suggesting that lacking financial resources was less of a barrier to OPPC use in the early COVID-19 period. Because older age and low income have been associated with eHealth activities, including OPPC, further investigations are warranted to confirm whether they remain in the extended COVID-19 period.

Notably, we observed different associations between depression and education with the use of OPPC among cancer survivors compared to adults without a cancer history. In our study, cancer survivors reporting depression as a chronic condition were less likely to use email/internet to communicate with providers than their counterparts during COVID-19. Prior studies either have not found associations [31] or have not assessed the associations of depression with OPPC [7,32]. However, depression was associated with the use of all 3 types of OPPC among adults without a history of cancer pre–COVID-19 and during COVID-19. The differing associations with OPPC among cancer survivors will need to be further investigated to determine whether our findings were specific to conditions in the early COVID-19 period that generated extreme mental distress. In addition, even though less educated adults without a history of cancer were less likely to use OPPC during COVID-19 and pre–COVID-19, these associations were not observed among cancer survivors in our study. In contrast to our findings, 2 prior studies (2003-2008 [31] and 2003-2018 [32]) have reported that highly educated cancer survivors are more likely to email providers [7]. Given the widespread use of email/internet, the education level may impact OPPC use less compared to other factors, such as access or eHealth literacy, that have been found to impact use more recently [55]. Therefore, our findings suggest that education level might not be a barrier to cancer survivors’ use of OPPC.

In this study, 16% of cancer survivors and 36% of US adults without a history of cancer reported no usual source of care, which was consistently associated with lower OPPC use among both cancer survivors and adults without a history of cancer before and during COVID-19. The likelihood of OPPC use among cancer survivors with a usual source of care appeared to be stronger during COVID-19. In addition, visiting the health provider’s office was strongly associated with EHR-based communications during the pandemic. One potential explanation could be that it would have been easier for those who had a usual source of care or recent office visits to connect with providers online than those without, particularly when in-person office visits were extremely limited under the stay-at-home order in 2020. Previous studies have not considered the usual source of care when assessing OPPC among cancer survivors [7,31,32]. However, it has been associated with OPPC in the general population [61]. To increase the usual source of care among cancer survivors, enhancing insurance coverage (eg, Medicaid expansion [51]) will need to be prioritized to improve health care access in underserved populations [62]. In addition, improving the perceived quality of care and physician trust [63,64] could improve health care–seeking behaviors [65,66].

Given that OPPC is a combination of health technology use and health care–seeking behavior, it requires a multifaceted approach to support it among cancer survivors. Prior studies have identified that health technology use is impacted by low digital device ownership, poor internet access, and lack of technical assistance [29,67,68] and health care seeking is lower among racial/ethnic minority populations and those with a poor patient-provider relationship [63,69]. Our study adds to this knowledge base by identifying vulnerable subgroups in OPPC. Interventions to improve OPPC should incorporate comprehensive and consistent health policies to cover diverse televisits (eg, audio-only calls, videoconferences), enhancing eHealth literacy, and increasing access to digital devices. Given that OPPC is technology-based communication, an effort to improve eHealth literacy among the targeted groups (eg, low socioeconomic status) is recommended, along with creating a technology-enabling environment [54]. One example of improving health literacy is the nationwide collaboration of the Adult Basic Education (ABE) network with community health organizations [70,71] by raising awareness of health literacy among ABE-registered low-literate individuals and implementing pilot projects into the targeted population via peers (eg, peer language navigators [72]). In addition, qualitative studies are suggested for a deeper understanding of barriers to and facilitators of OPPC in the vulnerable subgroups identified in this study.

### Limitations

This study has some limitations. First, because we used cross-sectional survey data, we could not determine the prospective and longitudinal associations with OPPC. Second, although the data used in this study were high-quality national survey data, they carry inevitable weaknesses originating from self-reporting and the possibilities of reporting bias (eg, communicated with providers via EHRs more than 12 months ago but reported it as within 12 months, intentionally or unintentionally). Third, due to the questionnaire time frame (in the past 12 months), it is possible that our outcome measurements during COVID-19 could have captured respondents’ behaviors before COVID-19. Fourth, the overall response rate of an average 33.0% during the study period could result in selection bias. However, HINTS applied full sampling weights and conducted imputation to minimize nonresponse. Fifth, the COVID-19 sample size was smaller than the pre–COVID-19 sample size (2017-2019) since the year 2020 was the only available data for COVID-19. Further, the HINTS 5 Cycle 4 questionnaires were administered and collected in the first half year of 2020 (February-June). Hence, we need to interpret the findings of this study from the context of the early COVID-19 period.

### Conclusion

Our findings suggest that cancer survivors who were older, had no usual source of care or health care office visits, had a low income, had public or no health insurance, were Hispanic, were unmarried, or reported depression were less likely to use OPPC during COVID-19, findings that differed from associations in adults without a history of cancer. As OPPC is increasingly becoming part of health care, we need to continue to evaluate disparities in its use in the extended COVID-19 period. Strategies to increase the use of OPPC include improvement in health policies to cover virtual visits, interventions to enhance eHealth literacy, and community-based or nationwide efforts to expand health technology access. Our findings identify vulnerable subgroups of cancer survivors with lower OPPC who can be targeted through multidimensional interventions to prevent further inequities.

## Appendix

Multimedia Appendix 1 Associations of sociodemographic and clinical factors with OPPC among noncancer populations pre–COVID-19 (2017-2019) and during COVID-19 (2020). OPPC: online patient-provider communication.

## Abbreviations

ABE: Adult Basic Education
CMS: Centers for Medicare and Medicaid Services
EHR: electronic health record
HINTS: Health Information National Trends Survey
IHS: Indian Health Services
OPPC: Online Patient-Provider Communication
OR: odds ratio
VA: Veterans Affairs

## Data Availability

The data for this study are publicly available [73].
