# Supplementary material for: Factors Associated With Online Patient-Provider Communications Among Cancer Survivors in the United States During COVID-19: Cross-sectional Study
Source: JMIR Cancer. 2023 May 22;9:e44339. doi: 10.2196/44339 (PMC10208306; doi:10.2196/44339)
Supplement: Multimedia Appendix 1 [file cancer_v9i1e44339_app1.docx]

**Supplemental table 1. Associations of sociodemographic and clinical factors with OPPC among non-cancer populations pre-COVID (2017-2019) and COVID (2020)**

|  | pre-COVID (2017-2019)^a^ | | | COVID^a^ | | |
| --- | --- | --- | --- | --- | --- | --- |
|  | Email/Internet | Tablet/SmartP | EHR | Email/Internet | Tablet/SmartP | EHR |
|  | aOR^b^ (95%CI) | aOR^b^ (95%CI) | aOR^b^ (95%CI) | aOR^b^ (95%CI) | aOR^b^ (95%CI) | aOR^b^ (95%CI) |
|  | N=9,940 | N=9,452 | N=8,522 | N=3,122 | N=3.113 | N=3,097 |
| **Age (years)** |  |  |  |  |  |  |
| 18-34 | 2.77  (1.96-3.91)* | 2.56  (1.79-3.66)* | 2.25  (1.38-3.69)* | 3.28  (2.08-5.20)* | 3.20  (1.51-6.78)* | 2.37  (1.12-5.05)* |
| 35-49 | 2.83  (2.05-3.89)* | 2.50  (1.81-3.46)* | 2.36  (1.48-3.75)* | 3.66  (2.26-5.95)* | 3.75  (1.76-8.02)* | 2.66  (1.39-5.10)* |
| 50-64 | 2.17  (1.58-2.97)* | 1.86  (1.40-2.48)* | 1.76  (1.12-2.76)* | 2.43  (1.57-3.77)* | 2.61  (1.36-5.01)* | 2.11  (1.11-4.01)* |
| 65-74 | 1.54  (1.16-2.05)* | 1.52  (1.14-2.03)* | 1.73  (1.18-2.53)* | 2.24  (1.48-3.40)* | 2.34  (1.28-4.29)* | 2.06  (1.19-3.57)* |
| $\geq$75 | Reference | Reference | Reference | Reference | Reference | Reference |
| **Race/Ethnicity** |  |  |  |  |  |  |
| Non-Hispanic  White | Reference | Reference | Reference | Reference | Reference | Reference |
| Non-Hispanic  Black/African | 1.06  (0.84-1.33) | 1.22  (0.96-1.55) | 1.13  (0.83-1.54) | 1.26  (0.78-2.04) | 2.27  (1.54-3.34)* | 1.45  (0.96-2.18) |
| Hispanic | 0.93  (0.75-1.16) | 0.98  (0.80-1.21) | 0.90  (0.69-1.17) | 1.38  (0.93-2.03) | 1.16  (0.80-1.66) | 0.90  (0.61-1.33) |
| Non-Hispanic  Asian | 1.37  (0.95-1.96) | 1.40  (1.01-1.95)* | 1.13  (0.77-1.66) | 0.92  (0.54-1.56) | 1.65  (0.99-2.74) | 2.46  (1.31-4.63)* |
| Others | 1.22  (0.80-1.86) | 1.06  (0.66-1.71) | 1.70  (0.96-3.03) | 1.85  (0.83-4.13) | 0.97  (0.55-1.71) | 0.56  (0.25-1.27) |
| **Education** |  |  |  |  |  |  |
| Less Than  High School | Reference | Reference | Reference | Reference | Reference | Reference |
| High School  Graduate | 1.76  (1.14-2.73)* | 1.94  (1.31-2.87)* | 1.52  (0.73-3.17) | 0.94  (0.41-2.15) | 1.26  (0.69-2.29) | 3.49  (1.39-8.74)* |
| Some College | 3.31  (2.18-5.03)* | 2.87  (1.95-4.22)* | 2.42  (1.17-5.01)* | 1.50  (0.69-3.26) | 1.80  (1.03-3.13)* | 3.59  (1.43-8.98)* |
| College Grad  or More | 4.76  (3.08-7.33)* | 3.72  (2.54-5.45)* | 3.14  (1.50-6.56)* | 3.26  (1.53-6.98)* | 2.65  (1.46-4.80)* | 6.47  (2.60-16.09)* |
| **Income** |  |  |  |  |  |  |
| < $20,000 | Reference | Reference | Reference | Reference | Reference | Reference |
| $20,000 to  < $35,000 | 1.01  (0.71-1.44) | 1.15  (0.79-1.66) | 1.03  (0.64-1.66) | 1.21  (0.76-1.94) | 0.99  (0.59-1.67) | 1.07  (0.52-2.18) |
| $35,000 to  < $50,000 | 1.38  (1.00-1.91)* | 1.16  (0.81-1.65) | 0.98  (0.61-1.57) | 1.38  (0.86-2.21) | 1.20  (0.70-2.06) | 2.04  (0.95-4.38) |
| $50,000 to  < $75,000 | 1.82  (1.29-2.56)* | 1.33  (0.94-1.89) | 1.64  (1.02-2.64)* | 1.55  (0.91-2.65) | 1.47  (0.87-2.47) | 1.68  (0.82-3.44) |
| $\geq$$75,000 | 2.60  (1.88-3.59)* | 1.37  (0.98-1.92) | 1.76  (1.12-2.78)* | 1.71  (1.09-2.68)* | 1.37  (0.80-2.33) | 1.81  (0.94-3.47) |
| **Marital status** |  |  |  |  |  |  |
| Married | 1.10  (0.96-1.26) | 1.19  (1.01-1.40)* | 1.13  (0.93-1.38) | 1.20  (0.92-1.57) | 1.61  (1.18-2.20)* | 1.40  (1.05-1.86)* |
| Not married | Reference | Reference | Reference | Reference | Reference | Reference |
| **Health insurance** |  |  |  |  |  |  |
| Private or by  employment | Reference | Reference | Reference | Reference | Reference | Reference |
| Medicare | 1.00  (0.76-1.33) | 0.75  (0.57-1.00) | 0.90  (0.61-1.33) | 0.93  (0.58-1.47) | 0.81  (0.47-1.39) | 0.96  (0.54-1.68) |
| Medicaid | 0.86  (0.63-1.18) | 1.03  (0.75-1.43) | 0.84  (0.56-1.25) | 0.98  (0.66-1.44) | 1.50  (1.01-2.21)* | 0.70  (0.40-1.21) |
| Tricare/VA/IHS | 1.14  (0.78-1.66) | 0.93  (0.65-1.31) | 1.02  (0.69-1.53) | 0.92  (0.60-1.42) | 1.11  (0.65-1.91) | 0.93  (0.54-1.63) |
| Others | 0.99  (0.73-1.34) | 1.00  (0.74-1.35) | 0.95  (0.61-1.49) | 0.51  (0.33-0.80)* | 1.06  (0.64-1.77) | 0.62  (0.34-1.11) |
| **Regular provider** |  |  |  |  |  |  |
| Yes | 1.66  (1.35-2.04)* | 1.58  (1.26-1.97)* | 2.33  (1.83-2.95)* | 1.76  (1.30-2.39)* | 1.68  (1.24-2.29)* | 2.29  (1.50-3.50)* |
| No | Reference | Reference | Reference | Reference | Reference | Reference |
| **Number of office visits (yearly)** |  |  |  |  |  |  |
| None | Reference | Reference | Reference | Reference | Reference | Reference |
| 1-4 times | 2.09  (1.64-1.66)* | 1.70  (1.34-2.17)* | 3.15  (2.12-4.68)* | 2.52  (1.67-3.81)* | 1.97  (1.35-2.86)* | 3.46  (1.85-6.48)* |
| 5-9 times | 3.18  (2.40-4.23)* | 2.60  (1.93-3.50)* | 4.16  (2.70-6.40)* | 4.62  (2.88-7.39)* | 3.57  (2.09-6.08)* | 6.30  (3.36-11.80)* |
| **Health status** |  |  |  |  |  |  |
| Excellent/good | 1.49  (1.16-1.91)* | 1.07  (0.84-1.36) | 1.06  (0.81-1.39) | 1.26  (0.81-1.98) | 1.10  (0.73-1.65) | 0.90  (0.55-1.46) |
| Fair/poor | Reference | Reference | Reference | Reference | Reference | Reference |
| **Medical condition** |  |  |  |  |  |  |
| Depression | 1.42  (1.17-1.74)* | 1.33  (1.07-1.65)* | 1.54  (1.23-1.92)* | 2.15  (1.55-2.98)* | 2.24  (1.57-3.21)* | 2.32  (1.52-3.55)* |
| No depression | Reference | Reference | Reference | Reference | Reference | Reference |
| **Mental health (past 2 weeks)** |  |  |  |  |  |  |
| Depression symptom | 1.06  (0.77-1.46) | 1.16  (0.82-1.64) | 1.00  (0.71-1.42) | 0.95  (0.61-1.47) | 0.84  (0.54-1.30) | 0.94  (0.61-1.46) |
| No symptom | Reference | Reference | Reference | Reference | Reference | Reference |
| Anxiety symptom | 1.12  (0.83-1.50) | 1.30  (0.91-1.86) | 0.86  (0.8-1.36) | 1.00  (0.57-1.73) | 0.73  (0.43-1.25) | 0.83  (0.51-1.36) |
| No symptom | Reference | Reference | Reference | Reference | Reference | Reference |

a. Total sample size: pre-COVID (N=10,124), COVID (N=3,168); b. Adjusted for all the variables in the Table; *P-value < 0.05; Abbreviations (SmartP: smartphone, EHR: Electronic Health Record, VA: Veterans Affairs, IHS: Indian Health Services)
